# Computerized videodefecography versus defecography: do we need radiographs?

**DOI:** 10.1590/S1516-31802005000300003

**Published:** 2005-05-02

**Authors:** Carlos Walter Sobrado, Carlos Eduardo Fonseca Pires, Sergio Eduardo Alonso Araújo, Edson Amaro, Angelita Habr-Gama, Desidério Roberto Kiss

**Keywords:** Defecography, Physiology, Anus, Radiation, Colorectal surgery, Defecografia, Fisiologia, Ânus, Radiação, Cirurgia colorectal

## Abstract

**CONTEXT AND OBJECTIVE:**

Defecography has been recognized as a valuable method for evaluating patients with evacuation disorders. It consists of the use of static radiography and fluoroscopy to record different situations within anorectal dynamics. Conventionally, rectal parameters are measured using radiograms. It is rare for fluoroscopy alone to be used. Computer software has been developed with the specific aim of calculating these measurements from digitized videotaped images obtained during fluoroscopy, without the need for radiographic film, thereby developing a computerized video-defecography method. The objective was thus to compare measurements obtained via computerized videodefecography with conventional measurements and to discuss the advantages of the new method.

**DESIGN AND SETTING:**

Prospective study at the radiology service of Hospital das Clínicas, Universidade de São Paulo.

**METHOD:**

Ten consecutive normal subjects underwent videodefecography. The anorectal angle, anorectal junction, puborectalis muscle length, anal canal length and degree of anal relaxation were obtained via the conventional method (using radiography film) and via computerized videodefecography using the ANGDIST software. Measurement and analysis of these parameters was performed by two independent physicians.

**RESULTS:**

Statistical analysis confirmed that the measurements obtained through direct radiography film assessment and using digital image analysis (computerized videodefecography) were equivalent.

**CONCLUSIONS:**

Computerized videodefecogra-phy is equivalent to the traditional defecography examination. It has the advantage of offering reduced radiation exposure through saving on the use of radiography.

## RESUMO

**CONTEXTO E OBJETIVO:**

O exame de defecografia tem sido reconhecido como método valioso para avaliação de pacientes com distúrbios da evacuação. A defecografia consiste em registrar, por meio de fluoroscopia e radiografias estáticas, diferentes situações da dinâmica anorretal. No método convencional, as radiografias são utilizadas para o cálculo de parâmetros retais. É rara a utilização apenas de fluoroscopia. Um programa de computador foi desenvolvido para calcular esses parâmetros através da digitalização das imagens registradas em vídeo pela fluoroscopia, criando um método de videodefecografia computadorizada. Assim, o objetivo foi de comparar os valores de parâmetros da defecografia calculados pelo método computadorizado proposto com aqueles obtidos por método convencional e de discutir as vantagens do novo método.

**TIPO DE ESTUDO E LOCAL:**

Estudo prospectivo no serviço de Radiologia do Hospital das Clínicas da Universidade de São Paulo.

**MÉTODO:**

Dez indivíduos voluntários normais foram submetidos ao exame de defecografia, no qual foram obtidas, a partir do método convencional (por meio de radiografias) e do método computadorizado (videodefecografia computadorizada), as seguintes medidas: ângulo anorretal, junção anorretal, comprimento do músculo puborretal, comprimento do canal anal e grau de abertura do ânus em cada uma das fases do exame. As avaliações e a análise dos parâmetros defecográficos acima descritos foram realizadas por dois observadores médicos independentes.

**RESULTADOS:**

Os resultados obtidos, após análise estatística, comprovaram a equivalência do método da videodefecografia computadorizada comparado ao método convencional.

**CONCLUSÕES:**

A videodefecografia computadorizada é método equivalente ao método tradicional de defecografia que permite menor exposição do paciente à irradiação por dispensar o uso de radiografias

## INTRODUCTION

Defecography is a radiological evaluation of the anatomy and function of the anorectum and pelvic floor. Initially described by Walldén in 1952, it is recognized as a valuable diagnostic method for the evaluation of anorectal physiology and evacuation disorders.^1-3^

Techniques for the examination vary signifi cantly between medical institutions, although most physicians attempt to follow standards recommended by Mahieu et al.^4^ Rectal outlining with a thick barium paste is followed by fluoroscopy, and radiographs are usually acquired to register anorectal events during three phases: squeeze, strain and rest. During this examination, several measurements (anorectal angle, anorectal junction, puborectalis muscle length, anal canal length and degree of anal relaxation) are usually obtained through direct examination of the X-ray films.^5,6^

At our institution, the methodology of videotaping the fluoroscopic images is usually employed (videodefecography). After digitizing the image, computer software called ANGDIST is used for determining the measurement parameters. Hence, X-ray film is seldom obtained, and the technique is known as computerized videodefecography.

## OBJECTIVE

This study was undertaken to compare measurements obtained by direct observation of radiographs and through computerized image analysis (computerized videodefecography), in a prospective manner using independent observers.

## METHOD

The present study underwent evaluation and was approved by the ethics committees of the Department of Gastroenterology (Universidade de São Paulo) and our university hospital (Hospital das Clínicas, Faculdade de Medicina da Universidade de São Paulo). Full informed consent was obtained from all participants.

Ten asymptomatic individuals volunteered to undergo videodefecography. Six individuals were female. Their ages ranged from 20 to 47 years, with a mean of 25 (standard deviation = 2.9 years). These individuals had no history of previous pelvic or anorectal surgery, without complaints of constipation or incontinence, and they said that they were able to move their bowels every day without the aid of laxatives. They did not have any clinically detected anorectal disease and presented normal sigmoidoscopy.

All individuals underwent standard videodefecography examination performed by the same radiology team. The equipment was the CGR (Compagnie Générale de Radiologie) TILTIX, connected to a video monitor and a professional VHS hi-fi videotape recorder (Intermed Video Technologies Inc., model GXR-70U HI-FI). The composition and density of the barium paste utilized were the same for all individuals. The barium was injected until the patients said that they had the sensation of a full rectum (a volume of approximately 200 ml).^2^

The following measurements were taken:

- anorectal angle (ARA): angle between the axes of the anal canal and rectum (taken at the back of the rectal wall). This was calculated during the three phases of the examination: squeeze (ARA-s), rest (ARAr) and defecation (ARA-d).
- anorectal junction (ARJ): distance between the anorectal junction and a line drawn from the tip of the coccyx to the inferior border of the pubic bone. This was also obtained during the three phases (ARJ-s, ARJ-r and ARJ-d).
- C-line (C)/puborectalis muscle length: line drawn from the inferior border of the pubic bone to the point on the posterior rectal wall with greatest flexion due to puborectalis muscle contraction. C was obtained during the three phases (C-s, C-r, and C-d).
- anal canal length (ACL)
- anal canal relaxation (ACR)

For each individual examined, two independent observers calculated these variables: one observer using conventional radiographic proctograms (defecography) and the other using computerized image analysis.

During computerized videodefecography analysis, the ANGDIST computer software was used for determining these measurements from the digitized videotape images. This software is able to calculate distances and angles from any image (in the form of bitmap files), with proper calibration of the distances and pincushion distortion adjustment. The software is calibrated by measuring the image of a metal sphere of 1.9 cm in diameter that is positioned above the patient's pubis during the examination.

The ANGDIST software was created by the engineer Paulo Eduardo Pilon using C++ programming language, and it runs under the Windows^®^ operating system. Further information about this software may be obtained by getting in touch with its creator, via the e-mail address paulo.pilon@terra.com.br.

For statistical analysis, variance analysis (ANOVA) was used to compare differences between the mean values of measurements obtained via the two methods. The Spearman rank correlation (*r_s_*) was used to determine the degree of correlation between measurements obtained via the two methods. The significance level adopted was 5% (p = 0.05).

**Table 1:** Mean values and correlations of measurements obtained during conventional defecography (CD) and computerized videodefecography (CVD)

Parameters	mean value			correlation	
	CD*	CDV†	p	r†	p
**ARA-s**	75.5	76	0.947	0.84	0.012
**ARJ-s**	2.27	2.13	0.715	0.97	0.004
**C-s**	5.37	5.49	0.796	0.91	0.006
**ARA-r**	114.35	114.9	0.931	0.93	0.005
**ARJ-r**	3.7	3.85	0.822	0.92	0.006
**C-r**	7.47	7.76	0.662	0.99	0.003
**ARA-d**	139.35	134.3	0.521	0.98	0.003
**ARJ-d**	4.67	4.66	0.985	0.88	0.009
**C-d**	8.7	8.93	0.700	0.99	0.003
**ACL**	1.87	1.91	0.765	0.84	0.012
**ACR**	1.19	1.25	0.623	0.92	0.006

* *ARA values in degrees. All other measurements in centimeters.*

† *Spearman rank correlation.*

*ARA = anorectal angle; ARJ = anorectal junction; C = puborectalis contraction; s = squeeze; r = rest; d = defecation; ACL = anal canal length; ACR = anal canal relaxation.*

## RESULTS

In this series, there was no significant difference between the mean values of the measurements obtained via conventional defecography and image analysis (computerized videodefecography), as presented in Table 1.

For all measurements, the Spearman correlation index was close to one, as shown in Table 1.

## DISCUSSION

Defecography has increasingly been recommended for the evaluation of anorectal function disorders, especially for constipation and incontinence.^6,7^ It is a noninvasive and well tolerated diagnostic tool that is capable of providing valuable information regarding patients' pelvic and anorectal physiology.^5^

Several measurements can be made via radiological examination, in particular the anorectal angle, anal canal length, degree of anal relaxation, puborectalis muscle length, perineal descent and degree of rectal evacuation. These measurements are usually obtained during defecography by means of direct calculations using radiographs taken following fluoroscopic scanning, since visual assessment of the fluoroscopy video monitor for this purpose is frequently hampered by image distortion.^2,6^

With the aim of overcoming this difficulty and doing away with the need for acquisition of radiographs, the ANGDIST computer software was developed. This program provides angle and distance calculations during the analysis of images obtained by digitizing analog fluoroscopic images recorded on videotape. Since ANGDIST uses independent vertical and horizontal calibration, image distortion is automatically corrected.^2^

The present study has demonstrated strong correlation between the measurements (ARA, ARJ and C during the three phases of the examination, and ACL and ACR) obtained via conventional analysis (defecography) and image analysis (computerized videodefecography). The two types of analysis were performed by different observers, with one blinded to the results of the other.

The degree of rectal evacuation was not evaluated in the present study, since the use of graphical computer analysis for obtaining this result had already begun at our institution in 1998.^2,8^

In spite of the report published by Mahieu et al.^4^ in 1984, regarding videotape recording of fluoroscopic images obtained during defecography (videodefecography), which showed that such a technique enables excellent dynamic evaluation of anorectal and pelvic function, the analysis of radiographic images remains the preferred method for the calculation of defecographic variables. Nevertheless, the acquisition of radiographs increases patients' exposure to radiation beyond the exposure needed for fluoroscopy recording.

Since fluoroscopic images can be recorded and stored, the development of a method that uses these images for the calculation of parameters, with no need for additional X-ray exposure, was highly desirable.^9-11^ After study of the feasibility of digital capture of videodefecography images, it became possible to devise computer software that was capable of defining measurement via image analysis.

Most individuals undergoing defecography are young people at reproductive age and, although the radiation dose during defecography largely remains within acceptable levels, computerized videodefecography does away with additional X-ray exposure.^3,11^ Moreover, the additional costs involved in performing videodefecography no longer represent a significant burden, since computer hardware is becoming more accessible.

## CONCLUSIONS

We conclude that videodefecography is feasible, provides defecographic parameters equivalent to conventional defecography and does away with the need for acquiring radiographs and the consequent additional exposure to radiation.
